# In-Situ Anaerobic Heating of Human Bones Probed by Neutron Diffraction

**DOI:** 10.1021/acs.analchem.2c04721

**Published:** 2023-01-13

**Authors:** Giulia Festa, Adriana P. Mamede, David Gonçalves, Eugénia Cunha, Winfried Kockelmann, Stewart F. Parker, Luís A. E. Batista de Carvalho, Maria Paula M. Marques

**Affiliations:** † CREF - Museo Storico della Fisica e Centro Studi e Ricerche “Enrico Fermi”, Via Panisperna 89a, Rome00184, Italy; ‡ Molecular Physical Chemistry R&D Unit, Department of Chemistry, University of Coimbra, Coimbra3004-535, Portugal; § Centre for Functional Ecology, Lab Forensic Anthropology, Department of Life Sciences, University of Coimbra, Coimbra3000-456, Portugal; ∥ Research Centre for Anthropology and Health (CIAS), University of Coimbra, Coimbra3000-456, Portugal; ⊥ Archaeosciences Lab, Directorate General Cultural Heritage (LARC/CIBIO/InBIO), Lisbon1300-418, Portugal; # Department of Life Sciences, University of Coimbra, Coimbra3000-456, Portugal; ∇ ISIS Pulsed Neutron and Muon Source, STFC Rutherford Appleton Laboratory, Chilton, DidcotOX11 0QX, United Kingdom

## Abstract

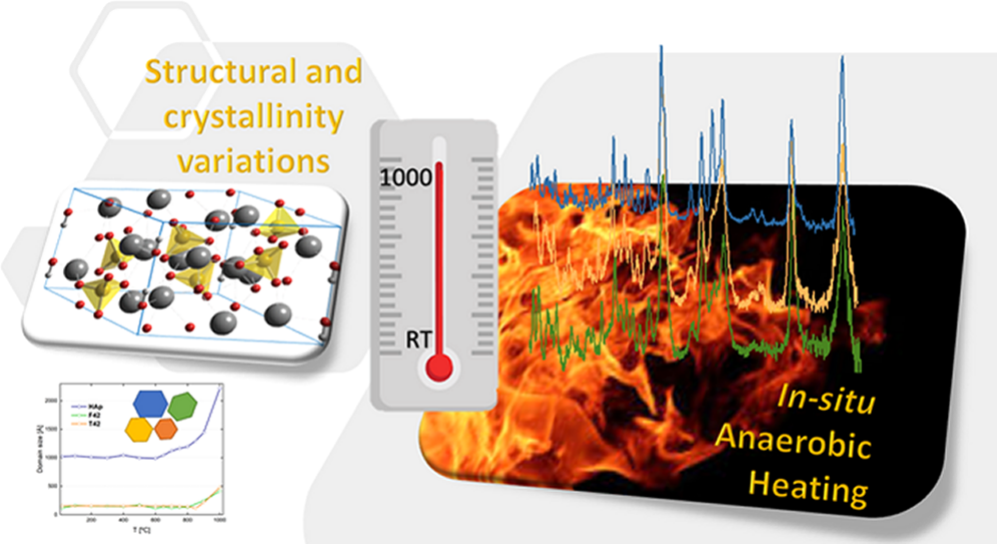

The first neutron diffraction study of in-situ anaerobic burning of human bones is reported, aiming at an interpretation of heat-induced changes in bone, which were previously detected by vibrational spectroscopy, including inelastic neutron scattering techniques. Structural and crystallinity variations were monitored in samples of the human femur and tibia, as well as a reference hydroxyapatite, upon heating under anaerobic conditions. Information on the structural reorganization of the bone matrix as a function of temperature, from room temperature to 1000 °C, was achieved. Noticeable crystallographic and domain size variations, together with O–H bond lengths and background variations, were detected. Above 700 °C, the inorganic bone matrix became highly symmetric, devoid of carbonates and organic constituents, while for the lower temperature range (<700 °C), a considerably lower crystallinity was observed. The present pilot study is expected to contribute to a better understanding of the heat-prompted changes in bone, which can be taken as biomarkers of the burning temperature. This information is paramount for bone analysis in forensic science as well as in archeology and may also have useful applications in other biomaterial studies.

## Introduction

Human bone is a heterogeneous tissue comprising type I collagen fibrils and several types of lipids, woven into a mineral matrix of hydroxyapatite (HAp, Ca_10_(PO_4_)_6_(OH)_2_) partially substituted by carbonates.^1−3^ Bone is known to experience morphological and structural changes upon burning processes. Neutron scattering techniques, both spectroscopy and diffraction, coupled with optical vibrational spectroscopy (Raman and Fourier transform infrared spectroscopy (FTIR)), have been successfully applied in the last few years to the study of burned human skeletal remains, with a view to assess heat-induced changes associated with alterations in the bone’s microcrystallinity.^4−13^ The examination of unburned skeletal remains is routinely carried out in forensic investigations to retrieve information on the circumstances of death, as well as on the biological profile and identity of the deceased, aiming at a positive identification. In the archeological context, the analysis of the remains is mainly devoted to the reconstruction of lifestyle from the skeleton. The analysis of bones affected by burning events, a common occurrence in forensic contexts, encounters challenging problems as heat induces significant physical and chemical changes in the skeleton, which interfere with the reliability of the available profiling methods. Thus, DNA identification is hindered by DNA destruction at high temperatures, and metric techniques (which are based on reference data from unburned bones) cannot be applied to burned samples.^5,14,15^ This provides the need to attain a thorough knowledge of the heat-elicited changes in human bones, leading to their prediction and quantification, aiming at the development of a reliable method for profiling burned human skeletal remains —both in forensic casework (e.g., victim identification in explosions, homicides, accidents, or domestic fires) and in archeological investigations.^16^

**Figure 1:**
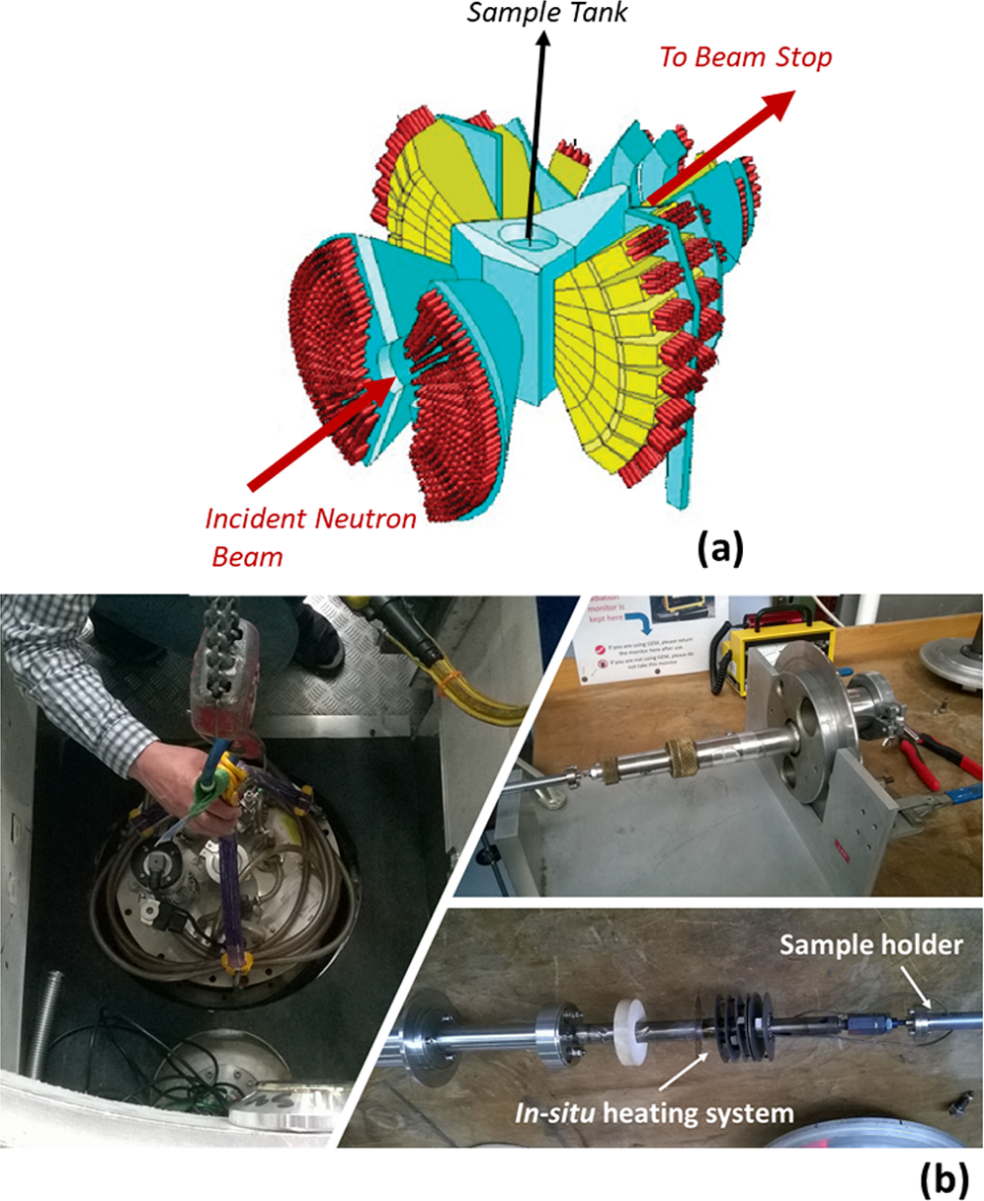
Experimental setup for the in-situ heating process of human bone samples, under controlled anaerobic conditions (vacuum), while measuring the corresponding neutron diffraction data at defined temperature points on the GEM neutron diffractometer; (a) layout of six ZnS/^6^Li scintillator detector arrays; (b) loading of the samples on GEM.

Neutron diffraction techniques are particularly suitable for probing burned bone,^3,12,17−19^ since they allow access, with high sensitivity, to the hydrogen atom positions in the inorganic matrix. This enables changes in the H-bond pattern within this crystalline framework to be determined. These are prone to occur upon heating events as previously suggested by neutron spectroscopy.^5,6,9,11−13^ The GEM diffractometer used in this study (at the ISIS Pulsed Neutron and Muon Source of the STFC Rutherford Appleton Laboratory, U.K.^20^) allows for the real-time monitoring of heat-induced changes as they occur—this is a longitudinal approach that probes variations within the same specimen during in-situ heating (Figure 1).

The present pilot study aims at a better understanding of the heat-prompted impact on human bones upon burning events under anaerobic conditions. This complements a previous neutron diffraction experiment on aerobically burned bone samples.^12^ Comparison between these two sets of data, for burning processes under distinct environmental conditions, will allow a comprehensive understanding of the effect of heating on human skeletal remains. In actual settings, both in forensic and archeological contexts, bone burning may have occurred, to some degree, under reducing conditions (absence of, or low, oxygen supply), namely, in fires within closed environments, cremations inside megalithic structures, or even during the manufacture of objects from skeletal remains. In archeological contexts, in particular, human burned bones found in megalithic tombs have often been interpreted as being the result of other than typical cremations of whole cadavers. Instead, some of them apparently resulted from different practices such as alternative funerary rites involving fire, sanitation procedures, or attempts to free some room in the chamber for new depositions.^21,22^ Given that such heat exposures were carried out in confined spaces, conditions close to anaerobic may have caused changes in the bone different from those usually observed in bones burned under aerobic conditions.^13,23^ The phases and compositions at particular temperatures were currently established for samples of human femur and tibia (osteometrically informative), upon a controlled anaerobic burning process at different maximum temperatures—from ca. 25 °C up to 1000 °C, the maximum value having been chosen according to temperatures typically reached in fire and explosion scenarios.^12^ Information on the structural organization of bone as a function of temperature was thus obtained, as well as on the rate of structural reorganization, through: (i) phase analysis—assessing changes in the bone’s crystal structure within the samples subject to increasing temperatures, while water, organic constituents (lipids and proteins), and carbonates were gradually driven out, thus characterizing the bone’s crystalline framework at well-defined temperatures; (ii) assessment of heat-induced variations in the structure, assuming that these do not occur instantaneously.

To the best of the authors’ knowledge, this is the first neutron diffraction study tackling the changes undergone by human bone during an in-situ heating process in the absence of oxygen, over a wide temperature range. The results thus gathered, combined with those previously obtained by optical (Raman and Fourier transform infrared (FTIR)) and neutron-based vibrational spectroscopy (inelastic neutron scattering (INS)), are intended to lay the basis for a more accurate analysis of human skeletal remains found in forensic scenarios (e.g., from terrorist attacks, aircraft accidents, or domestic fires) or in archeological sites, allowing to identify victims or obtain information on ancient civilizations (e.g., funerary practices)—regarding the conditions of burning (time, temperature, and oxygen availability) and the bone’s environmental setting.

## Experimental Section

### Materials

The bone samples studied in the present work were obtained from the human skeleton collection housed at the Laboratory of Forensic Anthropology of the University of Coimbra, which comprises unidentified skeletons from the cemetery of Capuchos (Santarém, Portugal).^24,25^ Femur (F) and tibia (T) bones from one skeleton were probed, hereafter denoted as F42 and T42, respectively. No replicates were analyzed due to the limited sample resources. The Ethics Committee of the Faculty of Medicine of the University of Coimbra authorized research on the CC_NI collection (reference number: CE_026.2016). Highly crystalline SRM 2910b calcium hydroxyapatite (HAp, Ca_10_(PO_4_)_6_(OH)_2_, Ca/P = 1.67, crystallinity index = 7.91) from NIST (Gaithersburg/MA) was used as a reference material.

### Sample Preparation and In-Situ Anaerobic Burning

Femur (F42) and tibia (T42) bone sections were cut (using a Dremel mini-saw electric tool), and contaminants from the outer layer were removed by gentle sanding. The fragments were ground, followed by sieving (mesh size of 400 μm), yielding 5–8 g of each sample. These were loaded into cylindrical vanadium containers of 11 mm diameter and 0.15 mm wall thickness, with a perforated lid to allow gas escape. Each container was inserted into a furnace inside the General Materials diffractometer (GEM)^26^ shown in Figure 1. The furnace allowed the collection of diffraction patterns under vacuum (<10^–3^ mbar) and at temperatures up to 1000 °C by means of radiative heating with a vanadium foil element heater. With this setup, the samples (including reference HAp) were heated from room temperature (RT, ca. 20 °C) to a maximum of 1000 °C. Two thermocouples, attached to the top part of the sample container, were used to record and regulate the sample temperature. The temperature was held constant within 2 K during data collection. The wide temperature range probed allowed us to monitor different heat-degradation events: water loss, pyrolysis of the organic components, bone porosity changes, and crystallite size increase.^14^

### Neutron Diffraction Measurements

Time-of-flight (TOF) diffraction data were collected by the General Materials powder diffractometer (GEM)^26^ at the ISIS Pulsed Neutron and Muon Source^20^ of the STFC Rutherford Appleton Laboratory (U.K.), within the scattering angle range 8–170°. GEM uses a polychromatic beam with neutron wavelengths ranging from 0.2 to 3.5 Å and a beam size at the sample of 20 × 40 mm^2^ (width × height). The average counting time was 1 h per temperature point.

### Neutron Diffraction Data Analysis

The complete data set was normalized using the MANTID software package,^27^ with data from a solid V3%Nb rod collected under the same experimental conditions. This normalization procedure takes into account the spectral distribution of neutrons and provides a correction for efficiency variations of individual detector elements. The data focus provides a grouping of the diffraction data into six histograms, i.e., diffraction patterns corresponding to average 2θ angles between 10° (Hist#1) and 155° (Hist#6), with normalized counts versus d-spacing.

## Results and Discussion

**Figure 2:**
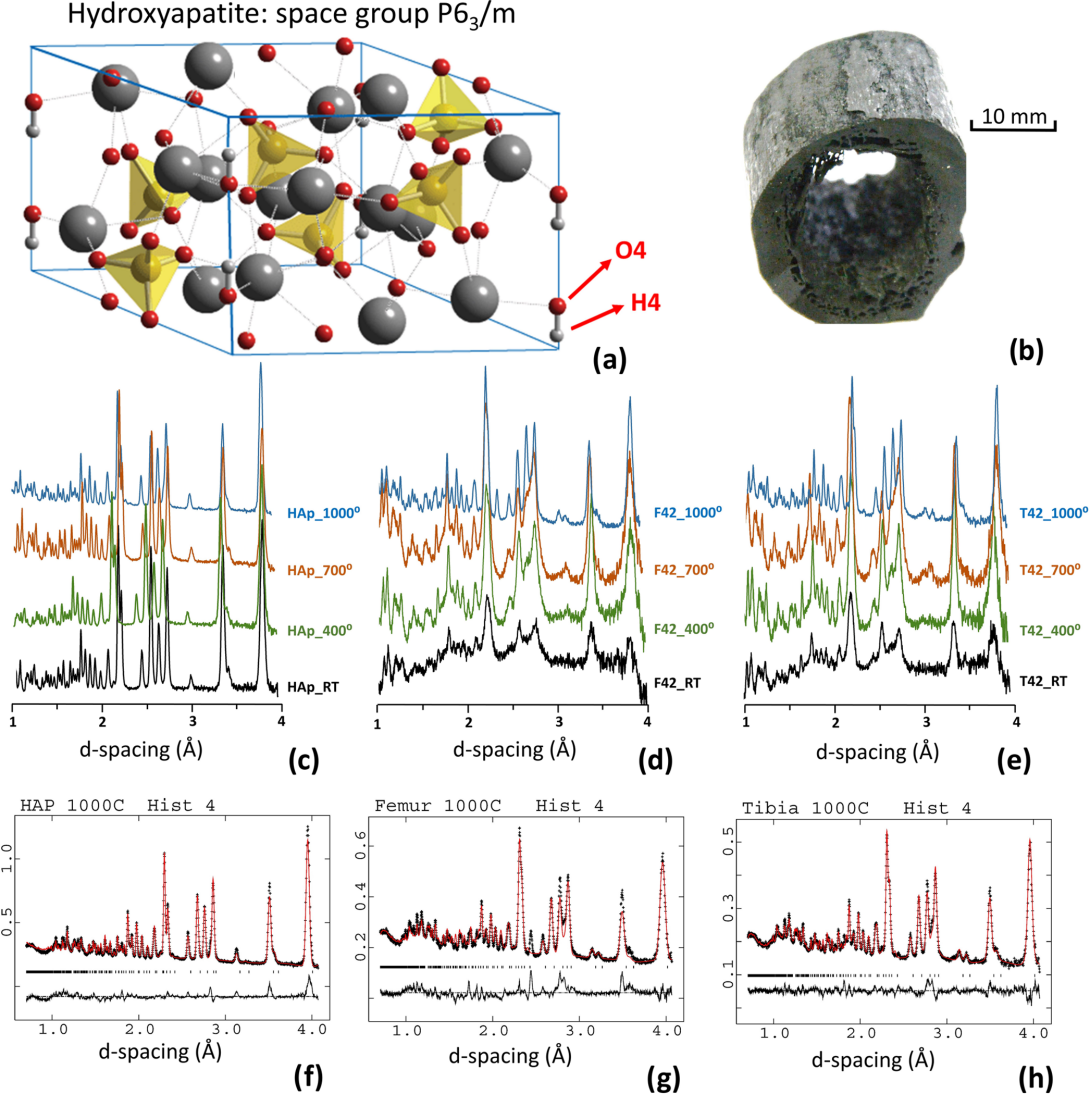
(a) Hydroxyapatite structure with Ca atoms displayed as gray spheres, hydrogen atoms in white, and P atoms inside oxygen (red spheres) tetrahedra. O4 and H4 atom positions at specific sites of the crystal structure are highlighted. (b) Section of human tibia burned at 1000 °C under anaerobic conditions. Neutron diffraction patterns at room temperature (RT) and after in-situ anaerobic burning at temperature points 400, 700, and 1000 °C, for (c) reference hydroxyapatite (HAp, SRM 2910b) data offset in y for clarity, (d) human femur (F42) data offset in *y* for clarity, (e) human tibia (T42) data offset for clarity. Bottom row: Rietveld refinement profiles of GEM histogram 4 at 1000 °C for: (f) HAp, (g) F42 and (h) T42.

Neutron diffraction patterns for the human bone samples (femur and tibia of sample SK42) and for a NIST standard reference material (SRM) hydroxyapatite (HAp) sample with in-situ anaerobic heating from room temperature (RT) up to a maximum of 1000 °C are presented in Figure 2 together with the typical structure of hydroxyapatite. The HAp reference sample shows a lower background in comparison to the femur (F42) and tibia (T42) samples because of the incoherent scattering from the organic components present in the real bone specimens. The background also varies as a function of temperature since the samples lose their organic components upon heating and develop into a higher crystallinity framework. Moreover, variations in the peak intensities and shapes are observed as a function of temperature, as well as increases in d-spacings of the diffraction peaks due to thermal expansion at higher temperatures.

For quantitative analysis, the Rietveld method was performed using the software GSAS-EXPGUI,^28,29^ using the reference structures of hexagonal hydroxyapatite (HAp) characterized by Kay et al.^30^ obtained from the FIZ Karlsruhe—Leibniz Institute for Information Infrastructure (ICSD). For hydroxyapatite, the space group *P*6_3_/*m* was used. The refinement procedure was developed first for the HAp reference data sets, which are characterized by a lower background and narrow peaks with fewer convergence problems, and then applied to the F42 and T42 samples. For the high-temperature data measured for HAp and the F42 and T42 samples, some parameters (namely OH positions) needed stronger constraints and were fixed to avoid overdetermination and correlations. All six GEM histograms were fitted with the GSAS time-of-flight profile function #2 (Ikeda Carpenter- pseudo-Voigt), and the following parameters were refined: scale factors and background polynomials for six histograms; lattice parameters; atom positions; O4 and H1 fractions; O–H bond lengths; Debye–Waller factors; diffractometer and profile parameters. Not to lose information, the Rietveld models were constrained only where needed to ensure convergence.

**Figure 3:**
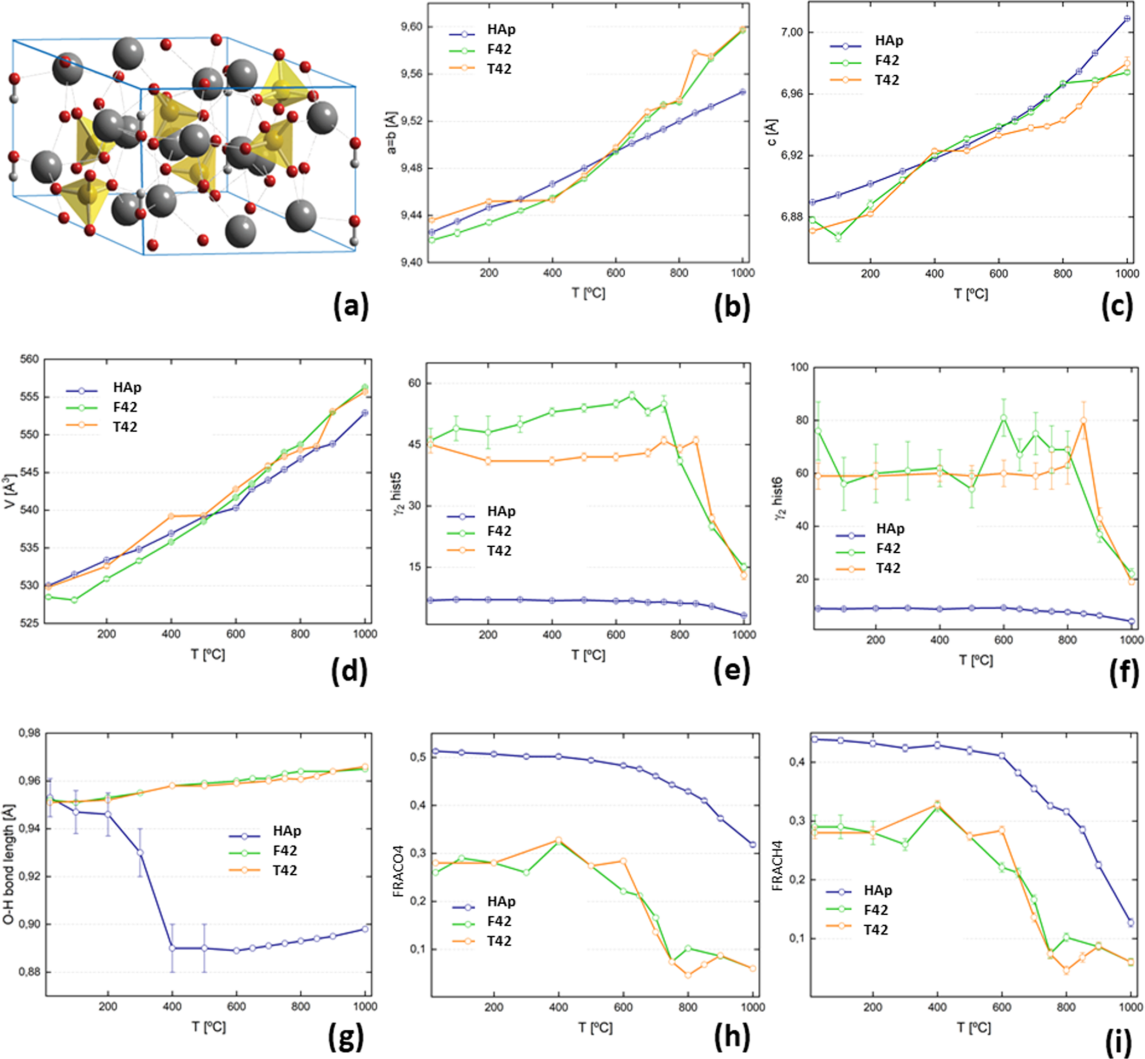
Plots of structure parameters during in-situ burning as a function of temperature, of hydroxyapatite, human femur, and human tibia: (a) hydroxyapatite structure; (b) *a* = *b* cell parameter; (c) *c* cell parameter; (d) unit cell volume; (e) γ_2_ value for histogram 5; (f) γ_2_ value for histogram 6; (g) OH bond length; (h) H4 fraction; and (i) O4 fraction. The lines through the data points are a guide to the eye.

**Figure 4:**
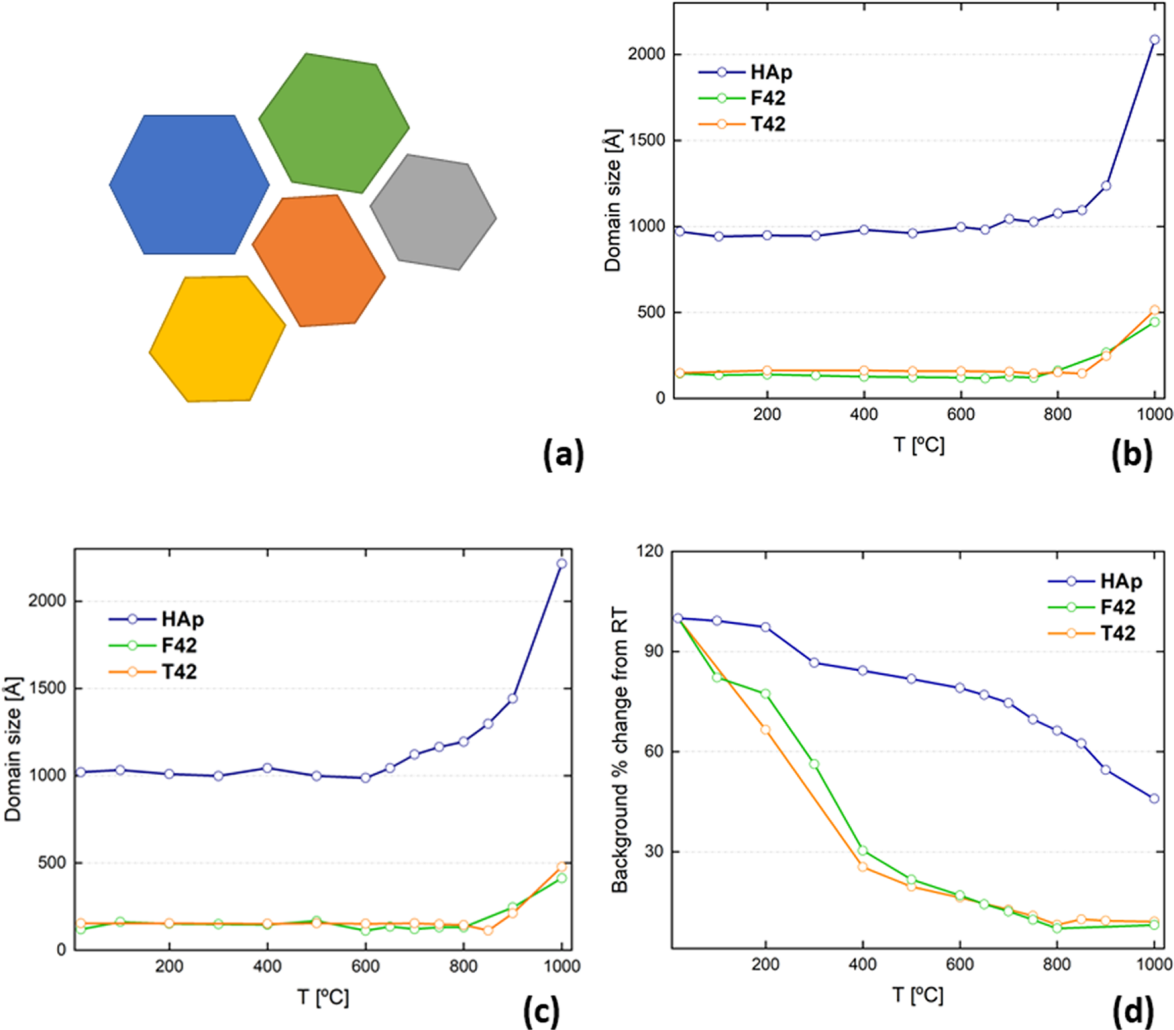
Crystallographic domain dimensions and background variations during in-situ burning as a function of temperature for hydroxyapatite, human femur, and human tibia: (a) schematic representation of the domains; (b) domain size - hist#5; (c) domain size - hist#6; (d) background percentage changes. The lines through the data points are a guide to the eye.

The results of the Rietveld refinement of the data are reported in Table 1. The crystal structure evolution of the hydroxyapatite phase in the samples, regarding the hydrogen atoms in the crystal structure, the hydrogen bonds, and the lattice parameters, was evaluated, starting from SRM HAp since the bone samples present the extra scattering from the organic components. Unit cell parameters (*a* = *b*, *c*) and the peak broadening parameter (γ_2_) for the investigated samples are reported together with the respective weighted profile *R*-factor (*R*_wp_). All the refined parameters in the data model, including all refined atomic positions, are tabulated for each temperature/sample in the SI. Information about the oxygen (O4) and hydrogen (H4) fractions and the O–H bond length are also shown in Table 1 and Figures 3 and 4. The unit cell parameters a = b and c, and consequently the cell volume, are found to increase as a function of temperature. The curves for the unit cell volumes (Figure 3d) indicate a similar positive thermal expansion for all three samples. This explains the previously reported reduction of the OH librational mode transition energy upon heating (e.g., 660–640 cm^–1^ from 200 to 650 °C, respectively).^13,23^ Much broader peaks (γ_2_ values, Figure 3e,f) for the femur and tibia compared to the SRM material are attributed to smaller hydroxyapatite domain sizes. The burning process involves a loss of organic components and at the same time favors recrystallization of the hydroxyapatite structure, leading to a microcrystallinity increase at high temperatures (above 700 °C). The O–H bond length for the HAp standard decreases from RT up to 400 °C (Figure 3g).

**Table 1:**
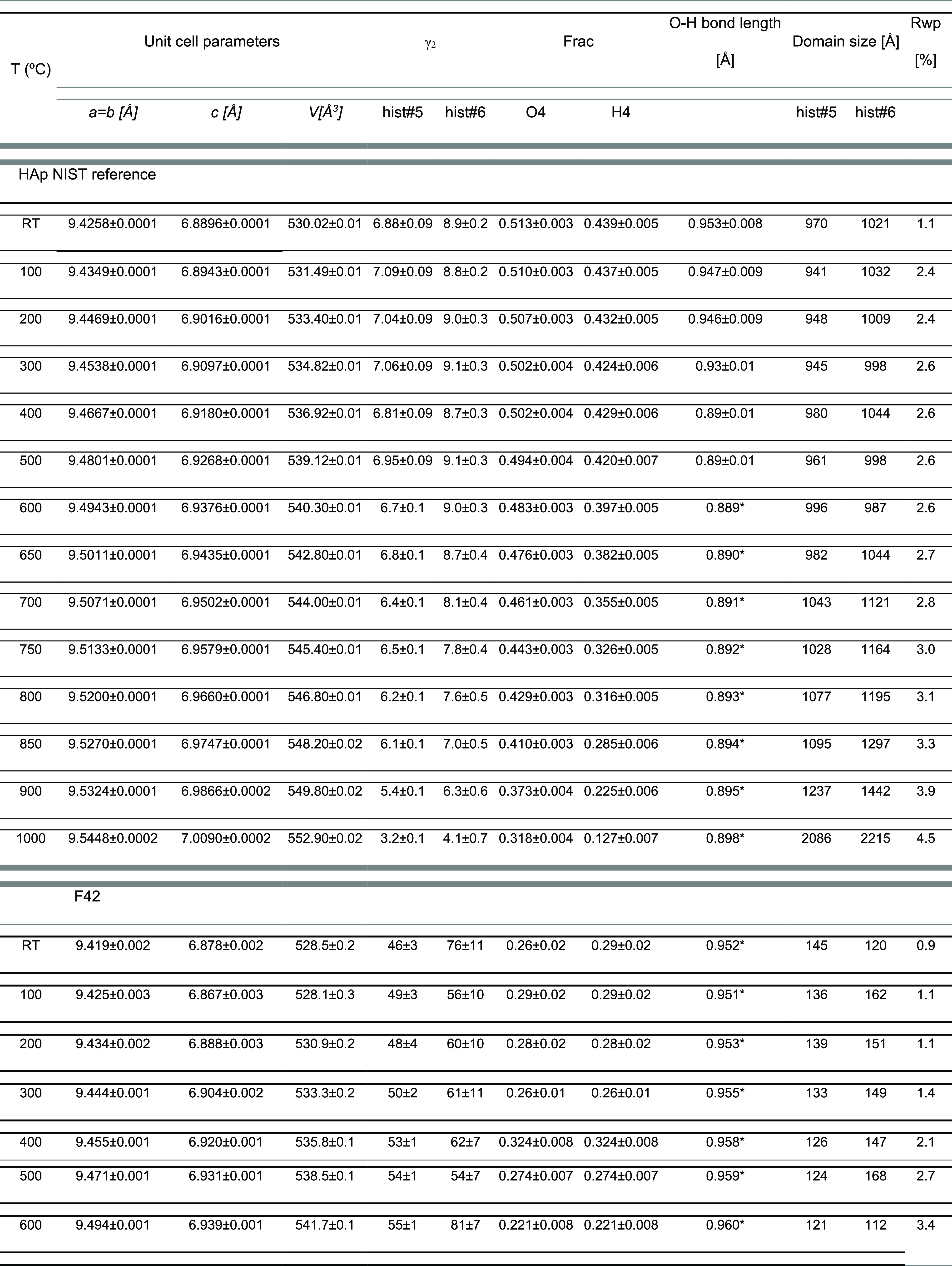

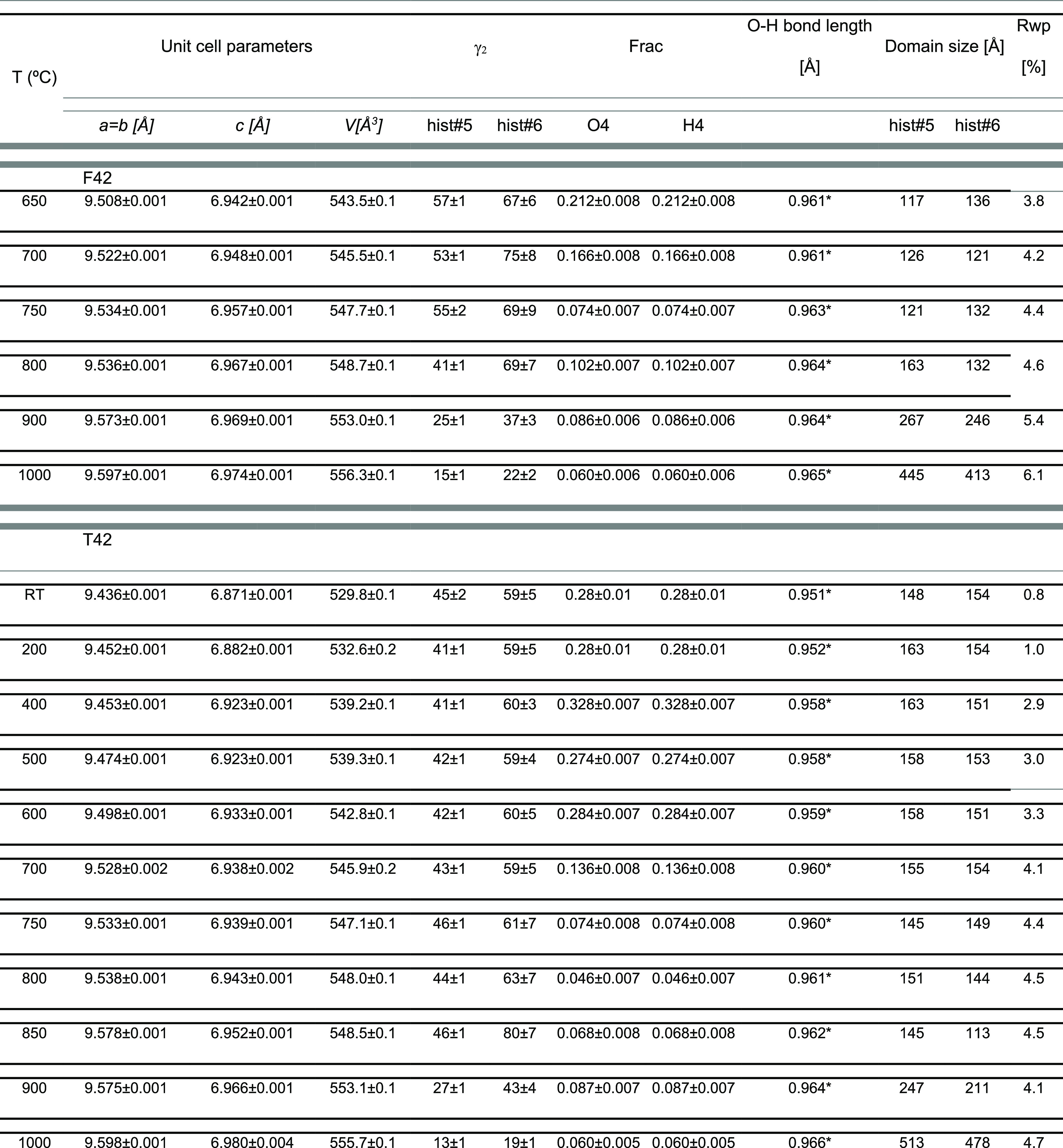
Structural Parameters Obtained via Rietveld Refinement for NIST SRM Hydroxyapatite, Human Femur and Tibia Samples (F42 and T42) Burned Anaerobically from Room Temperature (RT) to 1000 °C While Collecting Diffraction Data on GEM^a^

a Domain size parameters as a function of temperature are also reported. Oxygen (O4) and hydrogen (H4) fractions; O–H bond length; weighted profile *R*-factor (*R*_wp_) obtained via Rietveld refinement for all bone samples.

b Fixed OH positions.

Unfortunately, the data do not allow the O and H positions of the OH bonds (O4 and H4, respectively) to be refined above 400 °C for the SRM and for the real bone samples, so the positions of O4 and H4 were fixed. Hence, Hap, F42, and T42 all show an apparent slight increase in the O–H bond length above 400 °C, reflecting the increasing unit cell parameters. On the other hand, the O4 and H4 fractions of F42 and T42 display a clear drop above 600 °C (Figure 3h,i), whereas for HAp, the O4 and H4 fractions decrease more gradually toward higher temperatures.

From the peak broadening parameter γ_2_ (obtained through Rietveld refinement), the size of the diffracting domain (in Å) was determined from
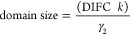
where DIFC is a GSAS diffractometer constant and *k* is the Scherrer constant, assumed to be 1. The domain sizes of the hydroxyapatite crystalline fractions within the samples were estimated for histograms 5 and 6 and are reported in Table 1 and Figure 4. It should be noted that the domain size for histogram 5 and histogram 6 should be the same and that histogram 6 provides the best estimates for the domain sizes due to the higher instrument resolution compared to histogram 5. The values for histogram 5 serve as a cross-check. The background variations in the RT diffraction profile are reported in percentage values in Figure 4d. The background percentage showed a higher decrease from RT to 400° for the two probed human bone samples (F42 and T42) as compared to the reference sample (HAp), while for higher temperatures, the trend was found to stabilize. This is suggested to be due to the loss of the organic fraction within the bone matrix during the burning process.^31^

The results depicted in Figure 4 reveal changes in crystallinity regions as a function of temperature—both for hydroxyapatite and the forensic samples F42 and T42—that can be attributed to an increasing structural organization when the temperature rises above 700–800 °C. Loss of hydrogen and organic components is evident from changes in the incoherent neutron scattering background (Figure 4d). The differences detected in the crystallographic parameters between the femur and tibia (F42 and T42) shown in Figures 3 and 4 are not significant. Among the investigated parameters that are related to the heat-induced changes in bone samples, irreversible parameters of the anaerobic burning process of human bones can be identified as biomarkers, such as H4 and O4 fractions, and domain sizes. Moreover, the diffraction background profile can be regarded as a further temperature marker since it relates to the loss of lipids, collagen, and water and decreases as a function of temperature (Figure 4d). Future perspectives in forensic studies are opened by the measure of O4–H4 fractions, which describe the position of the O and H atoms at specific sites in the crystal structure, and domain sizes at room temperature, recording temperature profiles of the parameters including the percentage scattering background to keep track of the burning process. Measurement of these parameters, especially when performed against a reference hydroxyapatite material, may indicate whether a sample had been burnt before and may reflect the temperature that was reached. It can be noted that reversible crystallographic parameters, such as the lattice parameters, are not useful as biomarkers of bone heat-elicited changes.^32^

## Conclusions

Neutron diffraction was applied to human bone samples burned under anaerobic conditions as an innovative approach for the elucidation of heat-elicited chemical and crystallinity changes. Noticeable structural variations were detected through the whole temperature range probed (from RT to 1000 °C). In particular, a clear enhancement of long-range order in terms of domain sizes was evidenced for the human femur and tibia specimens burned at temperatures above 700–800 °C, which is prompted by recrystallization of the hydroxyapatite structure and, at least in part, by the loss of bone’s organic constituents (collagen and lipids) as previously revealed by vibrational spectroscopy techniques (including INS).^5,6,13,23^ Irreversible structure parameters are identified as biomarkers of temperature changes elicited by the anaerobic burning of human bones such as H4 fractions and the domain sizes. Relative changes in hydrogen content can be analyzed, indicating loss of OH groups from the hydroxyapatite structure and loss of the organic components (lipids, collagen, and water). Based on these data, analysis strategies can be designed to help reconstruct the thermal history of forensic samples. Although the currently probed long bones are the most osteometrically informative in forensic, anthropological, and archeological sciences, the results cannot be generalized to all the long bones since only two samples (femur and tibia) from the same skeleton were analyzed. This is an understandable constraint in archeological studies, with the samples available for probing being often limited. The present work is therefore reported as a pilot study, expected to contribute to more reliable future identifications of human skeletal remains found in both forensic and archeological settings that have been subject to heating at unknown temperatures and environmental conditions.

## Data Availability

The data that support the findings of this study are available from the corresponding author upon reasonable request.

## Supporting Information Available

Structural data for the NIST HAp reference sample, human femur (F42) and human tibia (T42) as a function of temperature (PDF)

## Notes

The measurements were performed in accordance with current guidelines and regulations conducted within the authorizations obtained by the Laboratory of Forensic Anthropology of the University of Coimbra—Ethics Committee’s reference number: CE_026.2016.
