# Supplementary material for: In-Situ Anaerobic Heating of Human Bones Probed by Neutron Diffraction
Source: Anal Chem. 2023 Jan 13;95(4):2469–77. doi: 10.1021/acs.analchem.2c04721 (PMC9893223; doi:10.1021/acs.analchem.2c04721)
Supplement: Supplementary file 1 — ac2c04721_si_001.pdf [file ac2c04721_si_001.pdf]

SUPPORTING INFORMATION

In-situ anaerobic heating of human bones probed by neutron diffraction.

Giulia Festa[#], Adriana P. Mamede[$], David Gonçalves[%,^,&], Eugénia Cunha[%,~], Winfried Kockelmann[+], Stewart F. Parker[+*], Luís A.E. Batista de Carvalho[$] and Maria Paula M. Marques[$,~]

[#]CREF - Museo Storico della Fisica e Centro Studi e Ricerche "Enrico Fermi", Rome, Via Panisperna 89a, 00184, Italy

[$]University of Coimbra, Molecular Physical Chemistry R&D Unit, Department of Chemistry, Coimbra, 3004-535, Portugal

[%]Centre for Functional Ecology, Lab Forensic Anthropology, Department of Life Sciences, University of Coimbra, Coimbra, 3000-456, Portugal

[^]University of Coimbra, Research Centre for Anthropology and Health (CIAS), Coimbra, 3000-456, Portugal

[&]Archaeosciences Lab, Directorate General Cultural Heritage (LARC/CIBIO/InBIO), Lisbon, 1349-021, Portugal

[~]University of Coimbra, Department of Life Sciences, Coimbra, 3000-456, Portugal

[+]ISIS Pulsed Neutron and Muon Source, STFC Rutherford Appleton Laboratory, Chilton, Didcot, OX 11 0QX, United Kingdom

[*]corresponding author: stewart.parker@stfc.ac.uk

Contents

Structural data for the NIST HAp reference sample as a function of temperature — Pages 2 - 8

Structural data for the human femur (F42) sample as a function of temperature — Pages 9 - 15

Structural data for the human tibia (T42) sample as a function of temperature — Pages 16 - 21

ALL SAMPLES:

space_group_name $P6_3/m$ (176)

HAp NIST reference RT

cell_angle_alpha 90º
cell_angle_beta 90º
cell_angle_gamma 120º
cell_length_a = cell_length_b = (9.4258±0.0001) Å
cell_length_c = (6.8896±0.0001) Å
cell_volume = (530.02±0.01) Å
Equivalent positions:
(1) X Y Z (2) X-Y X 1/2+Z (3) -Y X-Y Z
(4) -X -Y 1/2+Z (5) Y-X -X Z (6) Y Y-X 1/2+Z
atom_site_label, atom_site_fract_x, atom_site_fract_y, atom_site_fract_z, frac

O1	0.32705(12)	0.48393(14)	0.2500000(0)	M(001)	O	1	1.000(0)
O2	0.58803(13)	0.46454(14)	0.2500000(0)	M(001)	O	2	1.000(0)
O3	0.34207(10)	0.25716(10)	0.07085(10)	1	O	3	1.000(0)
O4	0.0000000(0)	0.0000000(0)	0.1944(5)	3	O	4	0.513(3)
P1	0.39858(18)	0.36840(16)	0.2500000(0)	M(001)	P	5	1.000(0)
Ca1	0.3333000(0)	0.6667000(0)	0.00099(26)	3	CA	6	1.000(0)
Ca2	0.24619(20)	0.99388(20)	0.2500000(0)	M(001)	CA	7	1.000(0)
H1	0.0000000(0)	0.0000000(0)	0.0561(9)	3	H	8	0.439(5)

HAp NIST reference 100 °C

cell_angle_alpha 90º
cell_angle_beta 90º
cell_angle_gamma 120º
cell_length_a = cell_length_b = (9.4349±0.0001) Å
cell_length_c = (6.8943±0.0001) Å
cell_volume = (531.49±0.01) Å
Equivalent positions:
(1) X Y Z (2) X-Y X 1/2+Z (3) -Y X-Y Z
(4) -X -Y 1/2+Z (5) Y-X -X Z (6) Y Y-X 1/2+Z
atom_site_label, atom_site_fract_x, atom_site_fract_y, atom_site_fract_z, frac

O1	0.32712(13)	0.48392(14)	0.2500000(0)	M(001)	O	1	1.000(0)
O2	0.58783(14)	0.46462(14)	0.2500000(0)	M(001)	O	2	1.000(0)
O3	0.34223(10)	0.25737(11)	0.07103(11)	1	O	3	1.000(0)
O4	0.0000000(0)	0.0000000(0)	0.1945(5)	3	O	4	0.510(3)
P1	0.39858(18)	0.36843(16)	0.2500000(0)	M(001)	P	5	1.000(0)
Ca1	0.3333000(0)	0.6667000(0)	0.00129(27)	3	CA	6	1.000(0)
Ca2	0.24613(21)	0.99387(21)	0.2500000(0)	M(001)	CA	7	1.000(0)
H1	0.0000000(0)	0.0000000(0)	0.0571(9)	3	H	8	0.437(5)

HAp NIST reference 200 °C

cell_angle_alpha 90º
cell_angle_beta 90º
cell_angle_gamma 120º
cell_length_a = cell_length_b = (9.4469±0.0001) Å
cell_length_c = (6.9016±0.0001) Å
cell_volume = (533.40±0.01) Å
Equivalent positions:
(1) X Y Z (2) X-Y X 1/2+Z (3) -Y X-Y Z
(4) -X -Y 1/2+Z (5) Y-X -X Z (6) Y Y-X 1/2+Z
atom_site_label, atom_site_fract_x, atom_site_fract_y, atom_site_fract_z, frac

O1	0.32782(13)	0.48431(15)	0.2500000(0)	M(001)	O	1	1.000(0)
O2	0.58766(14)	0.46449(15)	0.2500000(0)	M(001)	O	2	1.000(0)
O3	0.34226(10)	0.25769(11)	0.07138(11)	1	O	3	1.000(0)
O4	0.000000(0)	0.000000(0)	0.1949(5)	3	O	4	0.507(3)
P1	0.39860(18)	0.36857(17)	0.2500000(0)	M(001)	P	5	1.000(0)
Ca1	0.3333000(0)	0.6667000(0)	0.00161(27)	3	CA	6	1.000(0)
Ca2	0.24582(21)	0.99396(21)	0.2500000(0)	M(001)	CA	7	1.000(0)
H1	0.0000000(0)	0.0000000(0)	0.0578(10)	3	H	8	0.432(5)

HAp NIST reference 300 °C

cell_angle_alpha 90º
cell_angle_beta 90º
cell_angle_gamma 120º
cell_length_a = cell_length_b = (9.4538±0.0001) Å
cell_length_c = (6.9097±0.0001) Å
cell_volume = (534.82±0.01) Å
Equivalent positions:
(1) X Y Z (2) X-Y X 1/2+Z (3) -Y X-Y Z
(4) -X -Y 1/2+Z (5) Y-X -X Z (6) Y Y-X 1/2+Z
atom_site_label, atom_site_fract_x, atom_site_fract_y, atom_site_fract_z, frac

O1	0.32819(14)	0.48455(15)	0.2500000(0)	M(001)	O	1	1.000(0)
O2	0.58714(15)	0.46433(15)	0.2500000(0)	M(001)	O	2	1.000(0)
O3	0.34233(11)	0.25798(11)	0.07148(11)	1	O	3	1.000(0)
O4	0.0000000(0)	0.0000000(0)	0.1952(6)	3	O	4	0.502(4)
P1	0.39826(19)	0.36860(17)	0.2500000(0)	M(001)	P	5	1.000(0)
Ca1	0.3333000(0)	0.6667000(0)	0.00123(29)	3	CA	6	1.000(0)
Ca2	0.24532(22)	0.99354(22)	0.2500000(0)	M(001)	CA	7	1.000(0)
H1	0.0000000(0)	0.0000000(0)	0.0608(11)	3	H	8	0.424(6)

HAp NIST reference 400 °C

cell_angle_alpha 90º
cell_angle_beta 90º
cell_angle_gamma 120º
cell_length_a = cell_length_b = (9.4667±0.0001) Å
cell_length_c = (6.9180±0.0001) Å
cell_volume = (536.92±0.01) Å
Equivalent positions:
(1) X Y Z (2) X-Y X 1/2+Z (3) -Y X-Y Z
(4) -X -Y 1/2+Z (5) Y-X -X Z (6) Y Y-X 1/2+Z
atom_site_label, atom_site_fract_x, atom_site_fract_y, atom_site_fract_z, frac

O1	0.32819(14)	0.48455(15)	0.2500000(0)	M(001)	O	1	1.000(0)
O2	0.58714(15)	0.46433(15)	0.2500000(0)	M(001)	O	2	1.000(0)
O3	0.34233(11)	0.25798(11)	0.07148(11)	1	O	3	1.000(0)
O4	0.0000000(0)	0.0000000(0)	0.1952(6)	3	O	4	0.502(4)
P1	0.39826(19)	0.36860(17)	0.2500000(0)	M(001)	P	5	1.000(0)
Ca1	0.3333000(0)	0.6667000(0)	0.00123(29)	3	CA	6	1.000(0)
Ca2	0.24532(22)	0.99354(22)	0.2500000(0)	M(001)	CA	7	1.000(0)
H1	0.0000000(0)	0.0000000(0)	0.0608(11)	3	H	8	0.429(6)

HAp NIST reference 500 °C

cell_angle_alpha 90º
cell_angle_beta 90º
cell_angle_gamma 120º
cell_length_a = cell_length_b = (9.4801±0.0001) Å
cell_length_c = (6.9268±0.0001) Å
cell_volume = (539.12±0.01) Å
Equivalent positions:
(1) X Y Z (2) X-Y X 1/2+Z (3) -Y X-Y Z
(4) -X -Y 1/2+Z (5) Y-X -X Z (6) Y Y-X 1/2+Z
atom_site_label, atom_site_fract_x, atom_site_fract_y, atom_site_fract_z, frac

O1	0.32972(15)	0.48541(16)	0.2500000(0)	M(001)	O	1	1.000(0)
O2	0.58621(16)	0.46372(16)	0.2500000(0)	M(001)	O	2	1.000(0)
O3	0.34211(12)	0.25879(12)	0.07202(12)	1	O	3	1.000(0)
O4	0.0000000(0)	0.0000000(0)	0.1946(7)	3	O	4	0.494(4)
P1	0.39755(20)	0.36875(19)	0.2500000(0)	M(001)	P	5	1.000(0)
Ca1	0.3333000(0)	0.6667000(0)	0.00146(31)	3	CA	6	1.000(0)
Ca2	0.24480(23)	0.99356(24)	0.2500000(0)	M(001)	CA	7	1.000(0)
H1	0.0000000(0)	0.0000000(0)	0.0661(13)	3	H	8	0.420(7)

HAp NIST reference 600 °C

cell_angle_alpha 90º
cell_angle_beta 90º
cell_angle_gamma 120º
cell_length_a = cell_length_b = (9.4943±0.0001) Å
cell_length_c = (6.9376±0.0001) Å
cell_volume = (540.30±0.01) Å
Equivalent positions:
(1) X Y Z (2) X-Y X 1/2+Z (3) -Y X-Y Z
(4) -X -Y 1/2+Z (5) Y-X -X Z (6) Y Y-X 1/2+Z
atom_site_label, atom_site_fract_x, atom_site_fract_y, atom_site_fract_z, frac

O1	0.32940(15)	0.48495(16)	0.2500000(0)	M(001)	O	1	1.000(0)
O2	0.58626(16)	0.46344(16)	0.2500000(0)	M(001)	O	2	1.000(0)
O3	0.34236(12)	0.25876(12)	0.07192(12)	1	O	3	1.000(0)
O4	0.0000000(0)	0.0000000(0)	0.1945540(0)	3	O	4	0.483(3)
P1	0.39826(21)	0.36878(18)	0.2500000(0)	M(001)	P	5	1.000(0)
Ca1	0.3333000(0)	0.6667000(0)	0.00118(31)	3	CA	6	1.000(0)
Ca2	0.24542(23)	0.99385(24)	0.2500000(0)	M(001)	CA	7	1.000(0)
H1	0.0000000(0)	0.0000000(0)	0.0664030(0)	3	H	8	0.397(5)

HAp NIST reference 650 °C

cell_angle_alpha 90º
cell_angle_beta 90º
cell_angle_gamma 120º
cell_length_a = cell_length_b = (9.5011±0.0001) Å
cell_length_c = (6.9435±0.0001) Å
cell_volume = (542.80±0.01) Å
Equivalent positions:
(1) X Y Z (2) X-Y X 1/2+Z (3) -Y X-Y Z
(4) -X -Y 1/2+Z (5) Y-X -X Z (6) Y Y-X 1/2+Z
atom_site_label, atom_site_fract_x, atom_site_fract_y, atom_site_fract_z, frac

O1	0.33017(16)	0.48581(18)	0.2500000(0)	M(001)	O	1	1.000(0)
O2	0.58540(17)	0.46270(18)	0.2500000(0)	M(001)	O	2	1.000(0)
O3	0.34212(13)	0.25947(13)	0.07239(13)	1	O	3	1.000(0)
O4	0.0000000(0)	0.0000000(0)	0.1945540(0)	3	O	4	0.476(3)
P1	0.39689(23)	0.36832(20)	0.2500000(0)	M(001)	P	5	1.000(0)
Ca1	0.3333000(0)	0.6667000(0)	0.00137(34)	3	CA	6	1.000(0)
Ca2	0.24463(26)	0.99410(26)	0.2500000(0)	M(001)	CA	7	1.000(0)
H1	0.0000000(0)	0.0000000(0)	0.0664030(0)	3	H	8	0.382(5)

HAp NIST reference 700 °C

cell_angle_alpha 90º
cell_angle_beta 90º
cell_angle_gamma 120º
cell_length_a = cell_length_b = (9.5071±0.0001) Å
cell_length_c = (6.9502±0.0001) Å
cell_volume = (544.00±0.01) Å
Equivalent positions:
(1) X Y Z (2) X-Y X 1/2+Z (3) -Y X-Y Z
(4) -X -Y 1/2+Z (5) Y-X -X Z (6) Y Y-X 1/2+Z
atom_site_label, atom_site_fract_x, atom_site_fract_y, atom_site_fract_z, frac
O1 0.32991(17) 0.48556(18) 0.2500000(0) M(001) O 1 1.000(0)
O2 0.58491(18) 0.46198(19) 0.2500000(0) M(001) O 2 1.000(0)
O3 0.34212(13) 0.25965(13) 0.07250(13) 1 O 3 1.000(0)
O4 0.0000000(0) 0.0000000(0) 0.1945540(0) 3 O 4 0.461(3)
P1 0.39654(24) 0.36772(21) 0.2500000(0) M(001) P 5 1.000(0)
Ca1 0.3333000(0) 0.6667000(0) 0.0018(4) 3 CA 6 1.000(0)
Ca2 0.24385(27) 0.99398(27) 0.2500000(0) M(001) CA 7 1.000(0)
H1 0.0000000(0) 0.0000000(0) 0.0664030(0) 3 H 8 0.355(5)

HAp NIST reference 750 °C

cell_angle_alpha 90º
cell_angle_beta 90º
cell_angle_gamma 120º
cell_length_a = cell_length_b = (9.5133±0.0001) Å
cell_length_c = (6.9579±0.0001) Å
cell_volume = (545.40±0.01) Å
Equivalent positions:
(1) X Y Z (2) X-Y X 1/2+Z (3) -Y X-Y Z
(4) -X -Y 1/2+Z (5) Y-X -X Z (6) Y Y-X 1/2+Z
atom_site_label, atom_site_fract_x, atom_site_fract_y, atom_site_fract_z, frac
O1 0.32986(18) 0.48591(20) 0.2500000(0) M(001) O 1 1.000(0)
O2 0.58443(20) 0.46126(20) 0.2500000(0) M(001) O 2 1.000(0)
O3 0.34213(14) 0.25999(14) 0.07290(14) 1 O 3 1.000(0)
O4 0.0000000(0) 0.0000000(0) 0.1945540(0) 3 O 4 0.443(3)
P1 0.39523(25) 0.36698(22) 0.2500000(0) M(001) P 5 1.000(0)
Ca1 0.3333000(0) 0.6667000(0) 0.0019(4) 3 CA 6 1.000(0)
Ca2 0.24312(28) 0.99443(29) 0.2500000(0) M(001) CA 7 1.000(0)
H1 0.0000000(0) 0.0000000(0) 0.0664030(0) 3 H 8 0.326(5)

***HAp NIST reference 800* °C**

cell_angle_alpha 90º
cell_angle_beta 90º
cell_angle_gamma 120º
cell_length_a = cell_length_b = (9.5200±0.0001) Å
cell_length_c = (6.9660±0.0001) Å
cell_volume = (546.80±0.01) Å
Equivalent positions:
(1) X Y Z (2) X-Y X 1/2+Z (3) -Y X-Y Z
(4) -X -Y 1/2+Z (5) Y-X -X Z (6) Y Y-X 1/2+Z
atom_site_label, atom_site_fract_x, atom_site_fract_y, atom_site_fract_z, frac

O1	0.32964(20)	0.48564(21)	0.2500000(0)	M(001)	O	1	1.000(0)
O2	0.58388(21)	0.46050(21)	0.2500000(0)	M(001)	O	2	1.000(0)
O3	0.34184(15)	0.26008(15)	0.07329(15)	1	O	3	1.000(0)
O4	0.0000000(0)	0.0000000(0)	0.1945540(0)	3	O	4	0.429(3)
P1	0.39386(26)	0.36616(23)	0.2500000(0)	M(001)	P	5	1.000(0)
Ca1	0.3333000(0)	0.6667000(0)	0.0019(4)	3	CA	6	1.000(0)
Ca2	0.24268(29)	0.99485(31)	0.2500000(0)	M(001)	CA	7	1.000(0)
H1	0.0000000(0)	0.0000000(0)	0.0664030(0)	3	H	8	0.316(5)

***HAp NIST reference 850* °C**

cell_angle_alpha 90º
cell_angle_beta 90º
cell_angle_gamma 120º
cell_length_a = cell_length_b = (9.5270±0.0001) Å
cell_length_c = (6.9747±0.0001) Å
cell_volume = (548.20±0.02) Å
Equivalent positions:
(1) X Y Z (2) X-Y X 1/2+Z (3) -Y X-Y Z
(4) -X -Y 1/2+Z (5) Y-X -X Z (6) Y Y-X 1/2+Z
atom_site_label, atom_site_fract_x, atom_site_fract_y, atom_site_fract_z, frac

O1	0.32972(21)	0.48561(22)	0.2500000(0)	M(001)	O	1	1.000(0)
O2	0.58296(22)	0.45957(22)	0.2500000(0)	M(001)	O	2	1.000(0)
O3	0.34199(16)	0.26039(16)	0.07336(16)	1	O	3	1.000(0)
O4	0.0000000(0)	0.0000000(0)	0.1945540(0)	3	O	4	0.410(3)
P1	0.39263(28)	0.36527(24)	0.2500000(0)	M(001)	P	5	1.000(0)
Ca1	0.3333000(0)	0.6667000(0)	0.0027(4)	3	CA	6	1.000(0)
Ca2	0.24214(31)	0.99507(32)	0.2500000(0)	M(001)	CA	7	1.000(0)
H1	0.0000000(0)	0.0000000(0)	0.0664030(0)	3	H	8	0.285(6)

HAp NIST reference 900 °C

cell_angle_alpha 90º
cell_angle_beta 90º
cell_angle_gamma 120º
cell_length_a = cell_length_b = (9.5324±0.0001) Å
cell_length_c = (6.9866±0.0002) Å
cell_volume = (549.80±0.02) Å
Equivalent positions:
(1) X Y Z (2) X-Y X 1/2+Z (3) -Y X-Y Z
(4) -X -Y 1/2+Z (5) Y-X -X Z (6) Y Y-X 1/2+Z
atom_site_label, atom_site_fract_x, atom_site_fract_y, atom_site_fract_z, frac
O1 0.32940(24) 0.48507(25) 0.2500000(0) M(001) O 1 1.000(0)
O2 0.58213(25) 0.45788(24) 0.2500000(0) M(001) O 2 1.000(0)
O3 0.34198(17) 0.26082(17) 0.07369(17) 1 O 3 1.000(0)
O4 0.0000000(0) 0.0000000(0) 0.1945540(0) 3 O 4 0.373(4)
P1 0.38990(30) 0.36368(27) 0.2500000(0) M(001) P 5 1.000(0)
Ca1 0.3333000(0) 0.6667000(0) 0.0021(5) 3 CA 6 1.000(0)
Ca2 0.24116(33) 0.9959(4) 0.2500000(0) M(001) CA 7 1.000(0)
H1 0.0000000(0) 0.0000000(0) 0.0664030(0) 3 H 8 0.225(6)

HAp NIST reference 1000 °C

cell_angle_alpha 90º
cell_angle_beta 90º
cell_angle_gamma 120º
cell_length_a = cell_length_b = (9.5448±0.0002) Å
cell_length_c = (7.0090±0.0002) Å
cell_volume = (552.90±0.02) Å
Equivalent positions:
(1) X Y Z (2) X-Y X 1/2+Z (3) -Y X-Y Z
(4) -X -Y 1/2+Z (5) Y-X -X Z (6) Y Y-X 1/2+Z
atom_site_label, atom_site_fract_x, atom_site_fract_y, atom_site_fract_z, frac
O1 0.32894(28) 0.48461(28) 0.2500000(0) M(001) O 1 1.000(0)
O2 0.57963(29) 0.45457(28) 0.2500000(0) M(001) O 2 1.000(0)
O3 0.34183(20) 0.26104(19) 0.07416(20) 1 O 3 1.000(0)
O4 0.0000000(0) 0.0000000(0) 0.1945540(0) 3 O 4 0.318(4)
P1 0.38730(34) 0.36189(31) 0.2500000(0) M(001) P 5 1.000(0)
Ca1 0.3333000(0) 0.6667000(0) 0.0039(5) 3 CA 6 1.000(0)
Ca2 0.2399(4) 0.9971(4) 0.2500000(0) M(001) CA 7 1.000(0)
H1 0.0000000(0) 0.0000000(0) 0.0664030(0) 3 H 8 0.127(7)

ALL SAMPLES:

space_group_name $P6_3/m$ (176)

F42 RT °C

cell_angle_alpha 90º
cell_angle_beta 90º
cell_angle_gamma 120º
cell_length_a = cell_length_b = (9.419±0.002) Å
cell_length_c = (6.878±0.002) Å
cell_volume = (528.5±0.2) Å
Equivalent positions:
(1) X Y Z (2) X-Y X 1/2+Z (3) -Y X-Y Z
(4) -X -Y 1/2+Z (5) Y-X -X Z (6) Y Y-X 1/2+Z
atom_site_label, atom_site_fract_x, atom_site_fract_y, atom_site_fract_z, frac

O1	0.3199(11)	0.4748(11)	0.2500000(0)	M(001)	O	1	1.000(0)
O2	0.5927(11)	0.4594(12)	0.2500000(0)	M(001)	O	2	1.000(0)
O3	0.3476(8)	0.2608(9)	0.0742(9)	1	O	3	1.000(0)
O4	0.0000000(0)	0.0000000(0)	0.1944220(0)	3	O	4	0.26(2)
P1	0.4091(16)	0.3759(15)	0.2500000(0)	M(001)	P	5	1.000(0)
Ca1	0.3333000(0)	0.6667000(0)	0.0062(25)	3	CA	6	1.000(0)
Ca2	0.2414(16)	0.9974(22)	0.2500000(0)	M(001)	CA	7	1.000(0)
H1	0.0000000(0)	0.0000000(0)	0.0560520(0)	3	H	8	0.29(2)

F42 100 °C

cell_angle_alpha 90º
cell_angle_beta 90º
cell_angle_gamma 120º
cell_length_a = cell_length_b = (9.425±0.003) Å
cell_length_c = (6.867±0.003) Å
cell_volume = (528.1±0.3) Å
Equivalent positions:
(1) X Y Z (2) X-Y X 1/2+Z (3) -Y X-Y Z
(4) -X -Y 1/2+Z (5) Y-X -X Z (6) Y Y-X 1/2+Z atom_site_label, atom_site_fract_x, atom_site_fract_y, atom_site_fract_z, frac

O1	0.3201(7)	0.4730(9)	0.2500000(0)	M(001)	O	1	1.000(0)
O2	0.5916(9)	0.4627(10)	0.2500000(0)	M(001)	O	2	1.000(0)
O3	0.3522(6)	0.2614(8)	0.0729(8)	1	O	3	1.000(0)
O4	0.0000000(0)	0.0000000(0)	0.1944220(0)	3	O	4	0.29(2)
P1	0.3990(11)	0.3648(12)	0.2500000(0)	M(001)	P	5	1.000(0)
Ca1	0.3333000(0)	0.6667000(0)	0.0015(20)	3	CA	6	1.000(0)
Ca2	0.2409(14)	0.9904(14)	0.2500000(0)	M(001)	CA	7	1.000(0)
H1	0.0000000(0)	0.0000000(0)	0.0560520(0)	3	H	8	0.29(2)

F42 200 **°C**

cell_angle_alpha 90°
cell_angle_beta 90°
cell_angle_gamma 120°
cell_length_a = cell_length_b = (9.434±0.002) Å
cell_length_c = (6.888±0.003) Å
cell_volume = (530.9±0.2) Å
Equivalent positions:
(1) X Y Z (2) X-Y X 1/2+Z (3) -Y X-Y Z
(4) -X -Y 1/2+Z (5) Y-X -X Z (6) Y Y-X 1/2+Z
atom_site_label, atom_site_fract_x, atom_site_fract_y, atom_site_fract_z, frac

O1	0.3221(7)	0.4734(8)	0.2500000(0)	M(001)	O	1	1.000(0)
O2	0.5895(8)	0.4607(9)	0.2500000(0)	M(001)	O	2	1.000(0)
O3	0.3527(6)	0.2638(7)	0.0729(7)	1	O	3	1.000(0)
O4	0.0000000(0)	0.0000000(0)	0.1944220(0)	3	O	4	0.28(2)
P1	0.3994(10)	0.3656(11)	0.2500000(0)	M(001)	P	5	1.000(0)
Ca1	0.3333000(0)	0.6667000(0)	-0.0041(17)	3	CA	6	1.000(0)
Ca2	0.2427(13)	0.9900(12)	0.2500000(0)	M(001)	CA	7	1.000(0)
H1	0.0000000(0)	0.0000000(0)	0.0560520(0)	3	H	8	0.28(2)

F42 300 **°C**

cell_angle_alpha 90°
cell_angle_beta 90°
cell_angle_gamma 120°
cell_length_a = cell_length_b = (9.444±0.001) Å
cell_length_c = (6.904±0.002) Å
cell_volume = (533.3±0.2) Å
Equivalent positions:
(1) X Y Z (2) X-Y X 1/2+Z (3) -Y X-Y Z
(4) -X -Y 1/2+Z (5) Y-X -X Z (6) Y Y-X 1/2+Z
atom_site_label, atom_site_fract_x, atom_site_fract_y, atom_site_fract_z, frac

O1	0.3237(4)	0.4764(5)	0.2500000(0)	M(001)	O	1	1.000(0)
O2	0.5855(5)	0.4611(6)	0.2500000(0)	M(001)	O	2	1.000(0)
O3	0.3511(4)	0.2625(4)	0.0716(5)	1	O	3	1.000(0)
O4	0.0000000(0)	0.0000000(0)	0.1944220(0)	3	O	4	0.26(1)
P1	0.3993(7)	0.3669(7)	0.2500000(0)	M(001)	P	5	1.000(0)
Ca1	0.3333000(0)	0.6667000(0)	0.0001(12)	3	CA	6	1.000(0)
Ca2	0.2484(8)	0.9948(8)	0.2500000(0)	M(001)	CA	7	1.000(0)
H1	0.0000000(0)	0.0000000(0)	0.0560520(0)	3	H	8	0.26(1)

F42 400 **°C**

cell_angle_alpha 90º
cell_angle_beta 90º
cell_angle_gamma 120º
cell_length_a = cell_length_b = (9.455±0.001) Å
cell_length_c = (6.920±0.001) Å
cell_volume = (535.8±0.1) Å
Equivalent positions:
(1) X Y Z (2) X-Y X 1/2+Z (3) -Y X-Y Z
(4) -X -Y 1/2+Z (5) Y-X -X Z (6) Y Y-X 1/2+Z
atom_site_label, atom_site_fract_x, atom_site_fract_y, atom_site_fract_z, frac

O1	0.3251(4)	0.4812(4)	0.2500000(0)	M(001)	O	1	1.000(0)
O2	0.5893(4)	0.4573(4)	0.2500000(0)	M(001)	O	2	1.000(0)
O3	0.34655(33)	0.26248(35)	0.0717(4)	1	O	3	1.000(0)
O4	0.0000000(0)	0.0000000(0)	0.1944220(0)	3	O	4	0.324(8)
P1	0.3934(6)	0.3625(6)	0.2500000(0)	M(001)	P	5	1.000(0)
Ca1	0.3333000(0)	0.6667000(0)	0.0061(10)	3	CA	6	1.000(0)
Ca2	0.2442(7)	1.0000(8)	0.2500000(0)	M(001)	CA	7	1.000(0)
H1	0.0000000(0)	0.0000000(0)	0.0560520(0)	3	H	8	0.324(8)

F42 500 **°C**

cell_angle_alpha 90º
cell_angle_beta 90º
cell_angle_gamma 120º
cell_length_a = cell_length_b = (9.471±0.001) Å
cell_length_c = (6.931±0.001) Å
cell_volume = (538.5±0.1) Å
Equivalent positions:
(1) X Y Z (2) X-Y X 1/2+Z (3) -Y X-Y Z
(4) -X -Y 1/2+Z (5) Y-X -X Z (6) Y Y-X 1/2+Z
atom_site_label, atom_site_fract_x, atom_site_fract_y, atom_site_fract_z, frac

O1	0.3255(4)	0.4814(4)	0.2500000(0)	M(001)	O	1	1.000(0)
O2	0.5880(4)	0.4592(4)	0.2500000(0)	M(001)	O	2	1.000(0)
O3	0.34718(32)	0.26208(34)	0.07086(34)	1	O	3	1.000(0)
O4	0.0000000(0)	0.0000000(0)	0.1944220(0)	3	O	4	0.274(7)
P1	0.3955(6)	0.3633(6)	0.2500000(0)	M(001)	P	5	1.000(0)
Ca1	0.3333000(0)	0.6667000(0)	0.0047(10)	3	CA	6	1.000(0)
Ca2	0.2481(7)	0.9990(7)	0.2500000(0)	M(001)	CA	7	1.000(0)
H1	0.0000000(0)	0.0000000(0)	0.0560520(0)	3	H	8	0.274(7)

F42 600 **°C**

cell_angle_alpha 90º
cell_angle_beta 90º
cell_angle_gamma 120º
cell_length_a = cell_length_b = (9.494±0.001) Å
cell_length_c = (6.939±0.001) Å
cell_volume = (541.7±0.1) Å
Equivalent positions:
(1) X Y Z (2) X-Y X 1/2+Z (3) -Y X-Y Z
(4) -X -Y 1/2+Z (5) Y-X -X Z (6) Y Y-X 1/2+Z
atom_site_label, atom_site_fract_x, atom_site_fract_y, atom_site_fract_z, frac

O1	0.3256(4)	0.4811(4)	0.2500000(0)	M(001)	O	1	1.000(0)
O2	0.5902(5)	0.4561(5)	0.2500000(0)	M(001)	O	2	1.000(0)
O3	0.34796(34)	0.26385(35)	0.0713(4)	1	O	3	1.000(0)
O4	0.0000000(0)	0.0000000(0)	0.1944220(0)	3	O	4	0.221(8)
P1	0.3960(6)	0.3627(6)	0.2500000(0)	M(001)	P	5	1.000(0)
Ca1	0.3333000(0)	0.6667000(0)	0.0068(10)	3	CA	6	1.000(0)
Ca2	0.2445(7)	0.9989(8)	0.2500000(0)	M(001)	CA	7	1.000(0)
H1	0.0000000(0)	0.0000000(0)	0.0560520(0)	3	H	8	0.221(8)

F42 650 **°C**

cell_angle_alpha 90º
cell_angle_beta 90º
cell_angle_gamma 120º
cell_length_a = cell_length_b = (9.508±0.001) Å
cell_length_c = (6.942±0.001) Å
cell_volume = (543.5±0.1) Å
Equivalent positions:
(1) X Y Z (2) X-Y X 1/2+Z (3) -Y X-Y Z
(4) -X -Y 1/2+Z (5) Y-X -X Z (6) Y Y-X 1/2+Z
atom_site_label, atom_site_fract_x, atom_site_fract_y, atom_site_fract_z, frac

O1	0.3265(4)	0.4824(4)	0.2500000(0)	M(001)	O	1	1.000(0)
O2	0.5898(5)	0.4553(5)	0.2500000(0)	M(001)	O	2	1.000(0)
O3	0.34879(34)	0.26520(35)	0.0714(4)	1	O	3	1.000(0)
O4	0.0000000(0)	0.0000000(0)	0.1944220(0)	3	O	4	0.212(8)
P1	0.3955(6)	0.3632(6)	0.2500000(0)	M(001)	P	5	1.000(0)
Ca1	0.3333000(0)	0.6667000(0)	0.0089(10)	3	CA	6	1.000(0)
Ca2	0.2444(7)	0.9993(8)	0.2500000(0)	M(001)	CA	7	1.000(0)
H1	0.0000000(0)	0.0000000(0)	0.0560520(0)	3	H	8	0.212(8)

F42 700 **°C**

cell_angle_alpha 90º
cell_angle_beta 90º
cell_angle_gamma 120º
cell_length_a = cell_length_b = (9.522±0.001) Å
cell_length_c = (6.948±0.001) Å
cell_volume = (545.5±0.1) Å
Equivalent positions:
(1) X Y Z (2) X-Y X 1/2+Z (3) -Y X-Y Z
(4) -X -Y 1/2+Z (5) Y-X -X Z (6) Y Y-X 1/2+Z
atom_site_label, atom_site_fract_x, atom_site_fract_y, atom_site_fract_z, frac

O1	0.3261(4)	0.4818(4)	0.2500000(0)	M(001)	O	1	1.000(0)
O2	0.5928(5)	0.4547(5)	0.2500000(0)	M(001)	O	2	1.000(0)
O3	0.35076(35)	0.26758(35)	0.0711(4)	1	O	3	1.000(0)
O4	0.0000000(0)	0.0000000(0)	0.1944220(0)	3	O	4	0.166(8)
P1	0.3970(6)	0.3639(6)	0.2500000(0)	M(001)	P	5	1.000(0)
Ca1	0.3333000(0)	0.6667000(0)	0.0100(11)	3	CA	6	1.000(0)
Ca2	0.2444(7)	1.0001(8)	0.2500000(0)	M(001)	CA	7	1.000(0)
H1	0.0000000(0)	0.0000000(0)	0.0560520(0)	3	H	8	0.166(8)

F42 750 **°C**

cell_angle_alpha 90º
cell_angle_beta 90º
cell_angle_gamma 120º
cell_length_a = cell_length_b = (9.534±0.001) Å
cell_length_c = (6.957±0.001) Å
cell_volume = (547.7±0.1) Å
Equivalent positions:
(1) X Y Z (2) X-Y X 1/2+Z (3) -Y X-Y Z
(4) -X -Y 1/2+Z (5) Y-X -X Z (6) Y Y-X 1/2+Z
atom_site_label, atom_site_fract_x, atom_site_fract_y, atom_site_fract_z, frac

O1	0.3260(4)	0.4804(4)	0.2500000(0)	M(001)	O	1	1.000(0)
O2	0.5885(5)	0.4631(5)	0.2500000(0)	M(001)	O	2	1.000(0)
O3	0.35073(35)	0.26728(34)	0.0694(4)	1	O	3	1.000(0)
O4	0.0000000(0)	0.0000000(0)	0.1944220(0)	3	O	4	0.074(7)
P1	0.3984(6)	0.3635(6)	0.2500000(0)	M(001)	P	5	1.000(0)
Ca1	0.3333000(0)	0.6667000(0)	0.0054(11)	3	CA	6	1.000(0)
Ca2	0.2528(7)	0.9993(8)	0.2500000(0)	M(001)	CA	7	1.000(0)
H1	0.0000000(0)	0.0000000(0)	0.0560520(0)	3	H	8	0.074(7)

F42 800 °C

cell_angle_alpha 90º
cell_angle_beta 90º
cell_angle_gamma 120º
cell_length_a = cell_length_b = (9.536±0.001) Å
cell_length_c = (6.967±0.001) Å
cell_volume = (548.7±0.1) Å
Equivalent positions:
(1) X Y Z (2) X-Y X 1/2+Z (3) -Y X-Y Z
(4) -X -Y 1/2+Z (5) Y-X -X Z (6) Y Y-X 1/2+Z
atom_site_label, atom_site_fract_x, atom_site_fract_y, atom_site_fract_z, frac
O1 0.3269(4) 0.4819(4) 0.2500000(0) M(001) O 1 1.000(0)
O2 0.5880(4) 0.4641(5) 0.2500000(0) M(001) O 2 1.000(0)
O3 0.35015(33) 0.26751(33) 0.06876(34) 1 O 3 1.000(0)
O4 0.0000000(0) 0.0000000(0) 0.1944220(0) 3 O 4 0.102(7)
P1 0.3973(6) 0.3633(6) 0.2500000(0) M(001) P 5 1.000(0)
Ca1 0.3333000(0) 0.6667000(0) 0.0048(10) 3 CA 6 1.000(0)
Ca2 0.2518(7) 0.9987(7) 0.2500000(0) M(001) CA 7 1.000(0)
H1 0.0000000(0) 0.0000000(0) 0.0560520(0) 3 H 8 0.102(7)

F42 900 °C

cell_angle_alpha 90º
cell_angle_beta 90º
cell_angle_gamma 120º
cell_length_a = cell_length_b = (9.573±0.001) Å
cell_length_c = (6.969±0.001) Å
cell_volume = (553.0±0.1) Å
Equivalent positions:
(1) X Y Z (2) X-Y X 1/2+Z (3) -Y X-Y Z
(4) -X -Y 1/2+Z (5) Y-X -X Z (6) Y Y-X 1/2+Z
atom_site_label, atom_site_fract_x, atom_site_fract_y, atom_site_fract_z, frac
O1 0.3318(5) 0.4830(5) 0.2500000(0) M(001) O 1 1.000(0)
O2 0.5917(5) 0.4658(5) 0.2500000(0) M(001) O 2 1.000(0)
O3 0.34707(33) 0.26916(32) 0.07298(35) 1 O 3 1.000(0)
O4 0.0000000(0) 0.0000000(0) 0.1944220(0) 3 O 4 0.086(6)
P1 0.3950(6) 0.3666(6) 0.2500000(0) M(001) P 5 1.000(0)
Ca1 0.3333000(0) 0.6667000(0) 0.0028(10) 3 CA 6 1.000(0)
Ca2 0.2441(7) 0.9940(8) 0.2500000(0) M(001) CA 7 1.000(0)
H1 0.0000000(0) 0.0000000(0) 0.0560520(0) 3 H 8 0.086(6)

***F42 1000* °C**

cell_angle_alpha 90º
cell_angle_beta 90º
cell_angle_gamma 120º
cell_length_a = cell_length_b = (9.597±0.001) Å
cell_length_c = (6.974±0.001) Å
cell_volume = (556.3±0.1) Å
Equivalent positions:
(1) X Y Z (2) X-Y X 1/2+Z (3) -Y X-Y Z
(4) -X -Y 1/2+Z (5) Y-X -X Z (6) Y Y-X 1/2+Z
atom_site_label, atom_site_fract_x, atom_site_fract_y, atom_site_fract_z, frac

O1	0.3351(5)	0.4831(5)	0.2500000(0)	M(001)	O	1	1.000(0)
O2	0.5905(6)	0.4672(6)	0.2500000(0)	M(001)	O	2	1.000(0)
O3	0.3451(4)	0.26854(34)	0.0729(4)	1	O	3	1.000(0)
O4	0.0000000(0)	0.0000000(0)	0.1944220(0)	3	O	4	0.060(6)
P1	0.3938(7)	0.3671(6)	0.2500000(0)	M(001)	P	5	1.000(0)
Ca1	0.3333000(0)	0.6667000(0)	0.0009(11)	3	CA	6	1.000(0)
Ca2	0.2443(7)	0.9913(8)	0.2500000(0)	M(001)	CA	7	1.000(0)
H1	0.0000000(0)	0.0000000(0)	0.0560520(0)	3	H	8	0.060(6)

ALL SAMPLES:

space_group_name $P6_3/m$ (176)

T42 RT °C

cell_angle_alpha 90º
cell_angle_beta 90º
cell_angle_gamma 120º
cell_length_a = cell_length_b = (9.436±0.001) Å
cell_length_c = (6.871±0.001) Å
cell_volume = (529.8±0.1) Å
Equivalent positions:
(1) X Y Z (2) X-Y X 1/2+Z (3) -Y X-Y Z
(4) -X -Y 1/2+Z (5) Y-X -X Z (6) Y Y-X 1/2+Z
atom_site_label, atom_site_fract_x, atom_site_fract_y, atom_site_fract_z, frac

O1	0.3253(7)	0.4795(7)	0.2500000(0)	M(001)	O	1	1.000(0)
O2	0.5895(7)	0.4650(7)	0.2500000(0)	M(001)	O	2	1.000(0)
O3	0.3462(5)	0.2615(6)	0.0709(6)	1	O	3	1.000(0)
O4	0.0000000(0)	0.0000000(0)	0.1944220(0)	3	O	4	0.28(1)
P1	0.4034(10)	0.3715(10)	0.2500000(0)	M(001)	P	5	1.000(0)
Ca1	0.3333000(0)	0.6667000(0)	0.0012(16)	3	CA	6	1.000(0)
Ca2	0.2476(12)	0.9974(13)	0.2500000(0)	M(001)	CA	7	1.000(0)
H1	0.0000000(0)	0.0000000(0)	0.0560520(0)	3	H	8	0.28(1)

T42 200 °C

cell_angle_alpha 90º
cell_angle_beta 90º
cell_angle_gamma 120º
cell_length_a = cell_length_b = (9.452±0.001) Å
cell_length_c = (6.882±0.001) Å
cell_volume = (532.6±0.2) Å
Equivalent positions:
(1) X Y Z (2) X-Y X 1/2+Z (3) -Y X-Y Z
(4) -X -Y 1/2+Z (5) Y-X -X Z (6) Y Y-X 1/2+Z
atom_site_label, atom_site_fract_x, atom_site_fract_y, atom_site_fract_z, frac

O1	0.3272(7)	0.4796(7)	0.2500000(0)	M(001)	O	1	1.000(0)
O2	0.5871(7)	0.4651(7)	0.2500000(0)	M(001)	O	2	1.000(0)
O3	0.3440(5)	0.2616(6)	0.0709(6)	1	O	3	1.000(0)
O4	0.0000000(0)	0.0000000(0)	0.1944220(0)	3	O	4	0.28(1)
P1	0.4008(10)	0.3709(9)	0.2500000(0)	M(001)	P	5	1.000(0)
Ca1	0.3333000(0)	0.6667000(0)	-0.0016(15)	3	CA	6	1.000(0)
Ca2	0.2483(11)	0.9986(12)	0.2500000(0)	M(001)	CA	7	1.000(0)
H1	0.0000000(0)	0.0000000(0)	0.0560520(0)	3	H	8	0.28(1)

T42 400 °C

cell_angle_alpha 90º
cell_angle_beta 90º
cell_angle_gamma 120º
cell_length_a = cell_length_b = (9.453±0.001) Å
cell_length_c = (6.923±0.001) Å
cell_volume = (539.2±0.1) Å
Equivalent positions:
(1) X Y Z (2) X-Y X 1/2+Z (3) -Y X-Y Z
(4) -X -Y 1/2+Z (5) Y-X -X Z (6) Y Y-X 1/2+Z
atom_site_label, atom_site_fract_x, atom_site_fract_y, atom_site_fract_z, frac

O1	0.3265(4)	0.4807(4)	0.2500000(0)	M(001)	O	1	1.000(0)
O2	0.5893(4)	0.4609(4)	0.2500000(0)	M(001)	O	2	1.000(0)
O3	0.34214(33)	0.25877(34)	0.07349(35)	1	O	3	1.000(0)
O4	0.0000000(0)	0.0000000(0)	0.1944220(0)	3	O	4	0.328(7)
P1	0.3923(6)	0.3633(5)	0.2500000(0)	M(001)	P	5	1.000(0)
Ca1	0.3333000(0)	0.6667000(0)	0.0014(9)	3	CA	6	1.000(0)
Ca2	0.2411(6)	0.9968(8)	0.2500000(0)	M(001)	CA	7	1.000(0)
H1	0.0000000(0)	0.0000000(0)	0.0560520(0)	3	H	8	0.328(7)

T42 500 °C

cell_angle_alpha 90º
cell_angle_beta 90º
cell_angle_gamma 120º
cell_length_a = cell_length_b = (9.474±0.001) Å
cell_length_c = (6.923±0.001) Å
cell_volume = (539.3±0.1) Å
Equivalent positions:
(1) X Y Z (2) X-Y X 1/2+Z (3) -Y X-Y Z
(4) -X -Y 1/2+Z (5) Y-X -X Z (6) Y Y-X 1/2+Z
atom_site_label, atom_site_fract_x, atom_site_fract_y, atom_site_fract_z, frac

O1	0.3265(4)	0.4807(4)	0.2500000(0)	M(001)	O	1	1.000(0)
O2	0.5893(4)	0.4609(4)	0.2500000(0)	M(001)	O	2	1.000(0)
O3	0.34214(33)	0.25877(34)	0.07349(35)	1	O	3	1.000(0)
O4	0.0000000(0)	0.0000000(0)	0.1944220(0)	3	O	4	0.274(7)
P1	0.3923(6)	0.3633(5)	0.2500000(0)	M(001)	P	5	1.000(0)
Ca1	0.3333000(0)	0.6667000(0)	0.0014(9)	3	CA	6	1.000(0)
Ca2	0.2411(6)	0.9968(8)	0.2500000(0)	M(001)	CA	7	1.000(0)
H1	0.0000000(0)	0.0000000(0)	0.0560520(0)	3	H	8	0.274(7)

T42 600 **°C**

cell_angle_alpha 90º
cell_angle_beta 90º
cell_angle_gamma 120º
cell_length_a = cell_length_b = (9.498±0.001) Å
cell_length_c = (6.933±0.001) Å
cell_volume = (542.8±0.1) Å
Equivalent positions:
(1) X Y Z (2) X-Y X 1/2+Z (3) -Y X-Y Z
(4) -X -Y 1/2+Z (5) Y-X -X Z (6) Y Y-X 1/2+Z
atom_site_label, atom_site_fract_x, atom_site_fract_y, atom_site_fract_z, frac
O1 0.3269(4) 0.4814(5) 0.2500000(0) M(001) O 1 1.000(0)
O2 0.5885(5) 0.4625(5) 0.2500000(0) M(001) O 2 1.000(0)
O3 0.34528(34) 0.26127(35) 0.0725(4) 1 O 3 1.000(0)
O4 0.0000000(0) 0.0000000(0) 0.1944220(0) 3 O 4 0.284(7)
P1 0.3962(6) 0.3645(6) 0.2500000(0) M(001) P 5 1.000(0)
Ca1 0.3333000(0) 0.6667000(0) 0.0015(10) 3 CA 6 1.000(0)
Ca2 0.2460(7) 0.9967(8) 0.2500000(0) M(001) CA 7 1.000(0)
H1 0.0000000(0) 0.0000000(0) 0.0560520(0) 3 H 8 0.284(7)

T42 700 **°C**

cell_angle_alpha 90º
cell_angle_beta 90º
cell_angle_gamma 120º
cell_length_a = cell_length_b = (9.528±0.002) Å
cell_length_c = (6.938±0.002) Å
cell_volume = (545.9±0.2) Å
Equivalent positions:
(1) X Y Z (2) X-Y X 1/2+Z (3) -Y X-Y Z
(4) -X -Y 1/2+Z (5) Y-X -X Z (6) Y Y-X 1/2+Z
atom_site_label, atom_site_fract_x, atom_site_fract_y, atom_site_fract_z, frac
O1 0.3269(5) 0.4814(5) 0.2500000(0) M(001) O 1 1.000(0)
O2 0.5922(5) 0.4580(5) 0.2500000(0) M(001) O 2 1.000(0)
O3 0.3479(4) 0.2655(4) 0.0735(4) 1 O 3 1.000(0)
O4 0.0000000(0) 0.0000000(0) 0.1944220(0) 3 O 4 0.136(8)
P1 0.3977(7) 0.3635(7) 0.2500000(0) M(001) P 5 1.000(0)
Ca1 0.3333000(0) 0.6667000(0) 0.0077(11) 3 CA 6 1.000(0)
Ca2 0.2445(7) 0.9989(9) 0.2500000(0) M(001) CA 7 1.000(0)
H1 0.0000000(0) 0.0000000(0) 0.0560520(0) 3 H 8 0.136(8)

T42 750 **°C**

cell_angle_alpha 90º
cell_angle_beta 90º
cell_angle_gamma 120º
cell_length_a = cell_length_b = (9.533±0.001) Å
cell_length_c = (6.939±0.001) Å
cell_volume = (547.1±0.1) Å
Equivalent positions:
(1) X Y Z (2) X-Y X 1/2+Z (3) -Y X-Y Z
(4) -X -Y 1/2+Z (5) Y-X -X Z (6) Y Y-X 1/2+Z
atom_site_label, atom_site_fract_x, atom_site_fract_y, atom_site_fract_z, frac

O1	0.3269(5)	0.4792(5)	0.2500000(0)	M(001)	O	1	1.000(0)
O2	0.5931(6)	0.4649(6)	0.2500000(0)	M(001)	O	2	1.000(0)
O3	0.3514(4)	0.2690(4)	0.0730(4)	1	O	3	1.000(0)
O4	0.0000000(0)	0.0000000(0)	0.1944220(0)	3	O	4	0.074(8)
P1	0.4019(8)	0.3650(8)	0.2500000(0)	M(001)	P	5	1.000(0)
Ca1	0.3333000(0)	0.6667000(0)	0.0054(13)	3	CA	6	1.000(0)
Ca2	0.2510(7)	1.0003(9)	0.2500000(0)	M(001)	CA	7	1.000(0)
H1	0.0000000(0)	0.0000000(0)	0.0560520(0)	3	H	8	0.074(8)

T42 800 **°C**

cell_angle_alpha 90º
cell_angle_beta 90º
cell_angle_gamma 120º
cell_length_a = cell_length_b = (9.538±0.001) Å
cell_length_c = (6.943±0.001) Å
cell_volume = (548.0±0.1) Å
Equivalent positions:
(1) X Y Z (2) X-Y X 1/2+Z (3) -Y X-Y Z
(4) -X -Y 1/2+Z (5) Y-X -X Z (6) Y Y-X 1/2+Z
atom_site_label, atom_site_fract_x, atom_site_fract_y, atom_site_fract_z, frac

O1	0.3274620(0)	0.4794350(0)	0.2500000(0)	M(001)	O	1	1.000(0)
O2	0.5914630(0)	0.4643810(0)	0.2500000(0)	M(001)	O	2	1.000(0)
O3	0.3504810(0)	0.2681880(0)	0.0728890(0)	1	O	3	1.000(0)
O4	0.0000000(0)	0.0000000(0)	0.1944220(0)	3	O	4	0.046(7)
P1	0.4003810(0)	0.3641230(0)	0.2500000(0)	M(001)	P	5	1.000(0)
Ca1	0.3333000(0)	0.6667000(0)	0.0053930(0)	3	CA	6	1.000(0)
Ca2	0.2514010(0)	0.9994940(0)	0.2500000(0)	M(001)	CA	7	1.000(0)
H1	0.0000000(0)	0.0000000(0)	0.0560520(0)	3	H	8	0.046(7)

T42 850 **°C**

cell_angle_alpha 90º
cell_angle_beta 90º
cell_angle_gamma 120º
cell_length_a = cell_length_b = (9.578±0.001) Å
cell_length_c = (6.952±0.001) Å
cell_volume = (548.5±0.1) Å
Equivalent positions:
(1) X Y Z (2) X-Y X 1/2+Z (3) -Y X-Y Z
(4) -X -Y 1/2+Z (5) Y-X -X Z (6) Y Y-X 1/2+Z
atom_site_label, atom_site_fract_x, atom_site_fract_y, atom_site_fract_z, frac

O1	0.3288(5)	0.4829(5)	0.2500000(0)	M(001)	O	1	1.000(0)
O2	0.5928(6)	0.4590(6)	0.2500000(0)	M(001)	O	2	1.000(0)
O3	0.3492(4)	0.2674(4)	0.0728(4)	1	O	3	1.000(0)
O4	0.0000000(0)	0.0000000(0)	0.1944220(0)	3	O	4	0.068(8)
P1	0.3965(7)	0.3651(7)	0.2500000(0)	M(001)	P	5	1.000(0)
Ca1	0.3333000(0)	0.6667000(0)	0.0080(12)	3	CA	6	1.000(0)
Ca2	0.2458(7)	0.9999(9)	0.2500000(0)	M(001)	CA	7	1.000(0)
H1	0.0000000(0)	0.0000000(0)	0.0560520(0)	3	H	8	0.068(8)

T42 900 **°C**

cell_angle_alpha 90º
cell_angle_beta 90º
cell_angle_gamma 120º
cell_length_a = cell_length_b = (9.575±0.001) Å
cell_length_c = (6.966±0.001) Å
cell_volume = (553.1±0.1) Å
Equivalent positions:
(1) X Y Z (2) X-Y X 1/2+Z (3) -Y X-Y Z
(4) -X -Y 1/2+Z (5) Y-X -X Z (6) Y Y-X 1/2+Z
atom_site_label, atom_site_fract_x, atom_site_fract_y, atom_site_fract_z, frac

O1	0.3303(5)	0.4849(5)	0.2500000(0)	M(001)	O	1	1.000(0)
O2	0.5907(5)	0.4601(5)	0.2500000(0)	M(001)	O	2	1.000(0)
O3	0.34612(35)	0.26702(34)	0.0748(4)	1	O	3	1.000(0)
O4	0.0000000(0)	0.0000000(0)	0.1944220(0)	3	O	4	0.087(7)
P1	0.3925(6)	0.3654(6)	0.2500000(0)	M(001)	P	5	1.000(0)
Ca1	0.3333000(0)	0.6667000(0)	0.0035(10)	3	CA	6	1.000(0)
Ca2	0.2398(7)	0.9949(8)	0.2500000(0)	M(001)	CA	7	1.000(0)
H1	0.0000000(0)	0.0000000(0)	0.0560520(0)	3	H	8	0.087(7)

***T42 1000* °C**

cell_angle_alpha 90º
cell_angle_beta 90º
cell_angle_gamma 120º
cell_length_a = cell_length_b = (9.598±0.001) Å
cell_length_c = (6.980±0.004) Å
cell_volume = (555.7±0.1) Å
Equivalent positions:
(1) X Y Z (2) X-Y X 1/2+Z (3) -Y X-Y Z
(4) -X -Y 1/2+Z (5) Y-X -X Z (6) Y Y-X 1/2+Z
atom_site_label, atom_site_fract_x, atom_site_fract_y, atom_site_fract_z, frac

O1	0.3334(4)	0.4842(4)	0.2500000(0)	M(001)	O	1	1.000(0)
O2	0.5890(5)	0.4647(5)	0.2500000(0)	M(001)	O	2	1.000(0)
O3	0.34395(29)	0.26694(28)	0.07431(31)	1	O	3	1.000(0)
O4	0.0000000(0)	0.0000000(0)	0.1944220(0)	3	O	4	0.060(5)
P1	0.3941(5)	0.3670(5)	0.2500000(0)	M(001)	P	5	1.000(0)
Ca1	0.3333000(0)	0.6667000(0)	0.0016(9)	3	CA	6	1.000(0)
Ca2	0.2443(6)	0.9939(6)	0.2500000(0)	M(001)	CA	7	1.000(0)
H1	0.0000000(0)	0.0000000(0)	0.0560520(0)	3	H	8	0.060(5)
